# Mitochondrial DNA polymorphisms, its copy number change and outcome in colorectal cancer

**DOI:** 10.1186/s13104-015-1250-5

**Published:** 2015-06-27

**Authors:** Asan Meera Sahib Haja Mohideen, Elizabeth Dicks, Patrick Parfrey, Roger Green, Sevtap Savas

**Affiliations:** Discipline of Genetics, Faculty of Medicine, Memorial University of Newfoundland, 300 Prince Philip Drive, HSC, Room H4333, St. John’s, NL A1B 3V6 Canada; Clinical Epidemiology Unit, Faculty of Medicine, Memorial University of Newfoundland, St. John’s, NL Canada; Discipline of Oncology, Faculty of Medicine, Memorial University of Newfoundland, St. John’s, NL Canada

**Keywords:** Mitochondrial DNA, Colorectal cancer, Prognosis, Polymorphisms, Mitochondrial DNA copy number change

## Abstract

**Background:**

Mitochondrion is a small organelle inside the eukaryotic cells. It has its own genome (mtDNA) and encodes for proteins that are critical for energy production and cellular metabolism. Mitochondrial dysfunctions have been implicated in cancer progression and may be related to poor prognosis in cancer patients. In this study we hypothesized that genetic variations in mtDNA are associated with clinical outcome in colorectal cancer patients.

**Methods:**

We tested the associations of six mtDNA polymorphisms [MitoT479C, MitoT491C, MitoT10035C, MitoA13781G, 10398 (A/G), and 16189 (T/C)] and the mtDNA copy number change with overall survival (OS) and disease-free survival (DFS) times. Two mtDNA polymorphisms were genotyped using the TaqMan^®^ SNP genotyping technique and the genotypes for the remaining four mtDNA polymorphisms were obtained by the Illumina^®^ HumanOmni1-Quad genome wide SNP genotyping platform in 536 patients. The mtDNA copy number change (in tumor tissues with respect to non-tumor tissues) was estimated using the quantitative real time polymerase chain reaction for 274 patients. Associations of these mtDNA variations with OS and DFS were tested using the Cox regression method.

**Results:**

In both univariate and multivariable analyses, none of the six mtDNA polymorphisms were associated with OS or DFS. 39.6 and 60.4% of the patients had increased and decreased mtDNA copy number in their tumor tissues when compared to their non-tumor rectum or colon tissues, respectively. However, in contrast to previous findings, the change in the mtDNA copy number was associated with neither OS nor DFS in our patient cohort.

**Conclusions:**

Our results suggest that the mitochondrial genetic markers investigated in this study are not associated with outcome in colorectal cancer.

**Electronic supplementary material:**

The online version of this article (doi:10.1186/s13104-015-1250-5) contains supplementary material, which is available to authorized users.

## Background

The uncontrolled proliferation of cells in colon or rectum is referred to as colorectal cancer. The western world has increased risk of developing this disease, which may be attributed to the lifestyle and dietary factors [1]. According to Global Cancer Statistics, worldwide colorectal cancer ranks as the fourth leading cause of cancer-mortality [2].

Mitochondrion is a small organelle in eukaryotic cells that exists in several copies. Mitochondria have important cellular functions such as energy production in the form of ATP through oxidative phosphorylation (OXPHOS) pathway, creation of reactive oxygen species, and initiation of apoptosis [3]. Mitochondrion has its own genome (mitochondrial DNA; mtDNA), which is a circular and double stranded DNA molecule approximately 16.5 kilo bases in length [4]. MtDNA has 37 genes out of which 22 code for tRNAs, two code for rRNAs, and 13 genes code for the respiratory chain proteins of the OXPHOS pathway. Genetic alterations in mtDNA such as point mutations, deletions and insertions are known to cause a metabolic change from OXPHOS to glycolysis pathway, which may facilitate cancer progression and resistance to chemotherapeutic drugs [5]. Since mitochondrial dysfunctions may modify cancer progression, genetic variations in mtDNA may have a prognostic role in cancer.

The main objective of this study was to analyze the genetic variations in mtDNA for their prognostic associations in colorectal cancer. For this purpose, we analyzed six single nucleotide polymorphisms (SNPs) in blood-extracted DNA and the mtDNA copy number change in tumor tissue with respect to matching non-tumor tissue as mitochondrial genetic markers. Among these six polymorphisms, the 10398 (A/G) and 16189 (T/C) polymorphisms were selected based on their potential biological consequences. The 10398 (A/G) polymorphism is a non-synonymous substitution located in the mitochondrial *ND*-*3* (NADH dehydrogenase 3) gene and has been shown to promote metastasis, resistance to apoptosis, and increased production of reactive oxygen species [6]. The 16189 (T/C) polymorphism, on the other hand, is located in the D-loop region of mtDNA, results in a heteroplasmic length variation, and has been hypothesized to affect the mtDNA replication and its cellular copy number [7, 8]. Four additional mtDNA polymorphisms were included in this study based on the availability of their genotype information obtained through a genome-wide SNP genotyping method. Of these, MitoT491C and MitoT479C polymorphisms are located in the mitochondrial D-loop region, the MitoA13781G polymorphism is located in the mitochondrial *ND*-*5* (NADH dehydrogenase 5) gene, and the MitoT10035 polymorphism is located in a tRNA gene that is specific for glycine codon. Biological consequences of these four mtDNA polymorphisms, if ever, are currently not known. In the present study, associations of these six polymorphisms with outcome were investigated in 536 colorectal cancer patients.

Changes in cellular copy number of mtDNA (either an increase or decrease when compared to other tissues) are observed in tumors, including colorectal cancer tumors [9–13]. This change is a surrogate marker for alterations in the number of mitochondria and thus its altered function. In this study in addition to the polymorphisms, association of the change in the mitochondrial DNA copy number in tumor tissue compared to matching non-tumor tissue of 274 colorectal cancer patients with outcome was also investigated.

## Methods

### Study design

This is a single center and retrospective study.

### Study cohorts

Genotyping of the six mtDNA polymorphisms and the mtDNA copy number analysis were performed on subsets of a colorectal cancer patient cohort recruited to the Newfoundland Colorectal Cancer Registry (NFCCR). The NFCCR cohort is a population-based and predominantly Caucasian cohort, which was described in detail previously [14, 15]. Briefly, patients in the NFCCR cohort were recruited between January 1999 and December 2003. Written consent was obtained from the patients or their family members. Patient blood samples were collected and used to extract the genomic DNA samples. Demographic, clinical and pathological characteristics of patients were collected from questionnaires, medical, pathological and other clinical records. Outcome status and dates of recurrence, metastasis or death were obtained from the clinical and hospital records, the Newfoundland Cancer Treatment and Research Foundation database, and by patient/family contact until April 2010 [16]. Tumor molecular characteristics, such as the microsatellite instability (MSI) and *BRAF1*-Val600Glu mutation status were determined previously using the tumor-extracted DNA [14]. The familial risk assessment was also performed previously as described by Green et al. [15].

NFCCR has 736 stage I–IV patients with collected prognostic data. For this study, the DNA samples (extracted from blood) of 536 of the patients were available; these patients were genotyped six mtDNA polymorphisms. In addition, out of 736 patients, 276 patients were investigated for the mtDNA copy number change based on the availability of their DNA samples extracted from both the tumor and non-tumor colon or rectum tissue; 210 of these patients were also investigated in the genotyping analysis. Baseline characteristics and the other relevant follow-up information of these two patient cohorts are summarized in Table 1.

**Table 1:** Baseline characteristics of patients included in the qPCR and the SNP genotyped cohorts

Variables	qPCR cohort	Polymorphism cohort
	n (%)	n (%)
Sex		
Female	116 (42.03)	208 (38.81)
Male	160 (57.97)	328 (61.19)
Location		
Colon	183 (66.3)	355 (66.23)
Rectum	93 (33.7)	181 (33.77)
Histology		
Non-mucinous	243 (88.04)	475 (88.62)
Mucinous	33 (11.96)	61 (11.38)
Stage		
I	31 (11.23)	98 (18.28)
II	94 (34.06)	207 (39.62)
III	101 (36.6)	178 (33.21)
IV	50 (18.11)	53 (9.89)
Grade		
Well/moderately differentiated	243 (88.04)	493 (91.98)
Poorly differentiated/undifferentiated	32 (11.6)	39 (7.28)
Unknown	1 (0.36)	4 (0.75)
Vascular invasion		
−	139 (50.36)	326 (60.82)
+	111 (40.22)	171 (31.9)
Unknown	26 (9.42)	39 (7.28)
Lymphatic invasion		
−	137 (49.64)	315 (58.77)
+	113 (40.94)	179 (33.4)
Unknown	26 (9.42)	42 (7.84)
Familial risk		
Low	142 (51.45)	256 (47.76)
High/intermediate	133 (48.19)	280 (52.24)
Unknown	1 (0.36)	
MSI status		
MSI-L/MSS	248 (89.86)	456 (85.07)
MSI-H	26 (9.42)	58 (10.82)
Unknown	2 (0.72)	22 (4.1)
*BRAF1* Val600Glu mutation		
−	219 (79.35)	436 (81.34)
+	35 (12.68)	49 (9.14)
Unknown	22 (7.97)	51 (9.51)
OS status		
Alive	141 (51.09)	353 (65.86)
Dead	134 (48.55)	182 (33.96)
Unknown	1 (0.36)	1 (0.19)
Median OS follow-up	6.2 years (range 0.036–10.88)	6.3 years (range 0.38–10.88)
DFS status		
Recurrence/metastasis/death (−)	126 (45.65)	322 (60.07)
Recurrence/metastasis/death (+)	149 (53.99)	213 (39.74)
Unknown	1 (0.36)	1 (0.19)
Median DFS follow-up	5.1 years (range 0.036–10.88)	5.98 years (range 0.22–10.88)
Median age	62.4 years (range 31.75–74.94)	61.3 years (range 20.7–74.98)

(+) presence, (−) absence, *DFS* disease free survival, *MSI-H* microsatellite instability-high, *MSI-L* microsatellite instability-low, *MSS* microsatellite stable, *n* number of patients, *OS* overall survival, *qPCR* quantitative PCR.

Prior ethics approval was obtained from the Human Research Ethics Board (HREB) of Memorial University to perform this study.

### SNP genotyping methods

Out of the six SNPs genotyped, two SNPs [16189 (T/C; rs55749223) and 10398 (A/G; rs2853826)] were genotyped using the TaqMan^®^ SNP genotyping technique. The primers and probes for the 16189 (T/C) polymorphism (Additional file 1: Table S1) were custom designed using the assay design tool of Applied Biosystem (https://www5.appliedbiosystems.com/tools/cadt/) and were purchased from the Applied Biosystems (USA). The primer and probe sequences for the 10398 (A/G) polymorphism were obtained from a published report [17] and were manufactured by the Integrated DNA Technologies (USA) and Applied Biosystems (USA), respectively.

For the genotyping experiments, we used 5 µl of PCR Master Mix (2X, Applied Biosystems, USA), 0.25 µl of 20X TaqMan^®^ Assay Mix, 3.75 µl of sterile water and 1 µl of DNA solution (4 ng/µl). The thermocycling conditions were as follow: Activation of AmpErase^®^ UNG at 50°C for 2 min, activation of AmpliTaq Gold polymerase at 95°C for 10 min, and 34 cycles of denaturation of DNA at 95°C for 15 s followed by primer annealing and extension at 60°C for 1 min, as recommended by the Applied Biosystems (USA). The experiments were run on a 7900HT Real Time Fast PCR system (Applied Biosystems, USA) and the results were analyzed using the Sequence Detection Software (SDS). The SDS genotype output data were also manually inspected by one of us (SS) to finalize the genotype data. For 16189 (T/C) and 10398 (A/G), 6.5 and 13.4% of the patient DNAs were genotyped twice, respectively; in all cases the genotypes obtained were identical.

The genotype data for the remaining four mtDNA polymorphisms (MitoT479C, rs41442247; MitoT491C, rs28625645; MitoT10035C, rs41347846; MitoA13781G, rs41358152) were obtained using a genome wide SNP genotyping technique (Illumina Human Omni1-Quad genomewide SNP genotyping platform) as part of another project of our team. These genotyping reactions were performed at an outsourced genotyping facility (Centrillion Genomics Services, CA, USA). Initially, genotypes for 25 mtDNA SNPs were available in the patient cohort. We found that seven of these SNPs were mono-allelic, some SNP-flanking sequences had a significant sequence homology with nuclear DNA sequences (thus it was not clear whether these genotypes were from mtDNA; n = 7), and seven SNPs had very low MAFs (<4%); these SNPs therefore were excluded from further analysis. As a result, the remaining four SNPs were included in the statistical analysis of this project. There were no duplicate samples genotyped using the Illumina^®^ HumanOmni-1 Quad SNP genotyping method; thus we were not able to assess the concordance rate for genotypes obtained for these four SNPs. To our best knowledge and based on the dbSNP database [18, 19], the nucleotide positions of these SNPs are annotated based on the Cambridge Reference Sequence (NC_001807.4), whereas the positions of the TaqMan^®^ genotyped polymorphisms are based on the revised Cambridge Reference Sequence (NC_012920.1). Throughout the manuscript we kept these positions as they are, but for the interested reader the nucleotide positions of the 16189 (T/C) and the 10398 (A/G) polymorphisms in the Cambridge Reference Sequence (NC_001807.4) are 16190 and 10399, respectively.

Genotype frequencies together with the successful genotyping rates for the six mtDNA polymorphisms investigated in this study are shown in Additional file 1: Table S1.

### Quantitative polymerase chain reaction (qPCR) for estimation of the mtDNA copy number ratio

The relative mtDNA copy number was estimated using a duplex quantitative polymerase chain reaction (qPCR). For qPCR reactions, the primer and probe sequences were obtained from a previously published literature report [20] and the primers and probes were manufactured by the Integrated DNA Technologies (USA) and Applied Biosystems (USA), respectively. In this duplex reaction, both a fragment of a nuclear gene (*FASLG;* surrogate for the nuclear DNA quantity) and a fragment of a mitochondrial gene (*ND*-*2* gene; surrogate for the mtDNA quantity) were amplified in the same reaction-well for each DNA sample using the 96-well fast PCR plates (Applied Biosystems, USA). The *FASLG* gene amplification data was used to normalize the *ND*-*2* gene amplification in each reaction to calculate the relative amplification of the mitochondrial DNA. For each patient, two separate qPCR reactions (one for the tumor extracted DNA and the other for the non-tumor tissue extracted DNA) were performed in triplicates. These two DNA samples for each patient were amplified in the same reaction plate using the same master reaction mix to minimize the inter-assay variability.

For qPCR analysis we used 5 µl PCR Master Mix (2X, Applied Biosystems, USA), 0.25 µl 20X TaqMan^®^ Assay Mix, 3.25 µl of sterile water and 1.5 µl of DNA solution (4 ng/µl). A series of optimization reactions were performed to identify the optimum concentration of primers and probes required to carry out the qPCR reaction. The contents of the 20X TaqMan^®^ Assay Mix used in this analysis are described in Additional file 1: Table S1. QPCR reactions were performed on a 7900HT Real Time Fast PCR system (Applied Biosystems, USA) and the thermocycling conditions for the qPCR were identical to the TaqMan^®^ SNP genotyping reaction explained in the previous section, except that the number of cycles was 36.

Once the qPCR reactions were completed, the amplification data was processed using the RQ Manager software (Applied Biosystems, USA). The baseline corrections were set manually for the *FASLG* and the *ND*-*2* amplifications separately and the amplification thresholds were either automatically or manually set based on the Applied Biosystems’ recommendations. For each qPCR reaction, the cycle number that bisects the threshold line (C_T_) was then exported and organized in Microsoft Excel© spread sheets.

For quality control measures, standard deviation (SD) among the C_T_ values of triplicate amplification of each DNA sample was computed using a Microsoft© Excel function. If the SD was >0.3 for the triplicates of a DNA sample, then the SD was reanalyzed by excluding one outlier C_T_ value. QPCR reactions were repeated for DNA samples that still had a C_T_ SD >0.3 after this step. Once all amplifications were completed, the average C_T_ value was calculated for each DNA sample and the mtDNA copy number ratio in tumor tissues with respect to non-tumor tissues was computed for each patient using the ∆∆C_T_ method [21]. Out of 276 patients included in this study, the mtDNA copy number ratio was obtained for 274 patients. For the primary analysis, if for a patient the ratio of mtDNA copy number in the tumor tissue to non-tumor tissue (mtDNA_T/N_) was >1, then we assumed an increase in mtDNA copy number in tumor tissue when compared to non-tumor tissue. If mtDNA_T/N_ was <1, then we assumed a decrease in mtDNA copy number in the tumor tissue when compared to non-tumor tissue. For the secondary and exploratory analysis, we considered the mtDNA_T/N_ <0.8 as a decrease and mtDNA_T/N_ >1.2 as an increase in a more stringent categorization of the relative mtDNA copy number. During this study, the mtDNA ratio values were rounded off to three decimal digits.

### Statistical analyses

Patient genotypes and the clinicopathological variables were coded in Microsoft^®^ Excel spread sheets prior to the statistical analysis. For six mtDNA polymorphisms, patients with the minor allele homoplasmy and major allele homoplasmy were categorized in two separate groups. Two patients, who were detected as heteroplasmic (i.e. containing the both alleles) in the case of 10398 (A/G) or 16189 (T/C) polymorphisms, were excluded from statistical analyses. For the mtDNA copy number analysis, patients with an increase in the mtDNA copy number in the tumor tissue relative to the non-tumor tissue were categorized together and were compared with patients with a decrease in the mtDNA copy number ratio. Clinicopathological features were also categorized, except age, which was analyzed as a continuous variable. Differences among the baseline characteristics of the patient cohorts were tested using the Chi square test for the categorical variables and the Mann–Whitney U test for the continuous variables. The possible associations between the SNP genotypes or mtDNA copy number change and the clinicopathological variables were tested by Chi square or Fisher’s exact test.

There were two measures of outcome used in the statistical analysis—overall survival (OS) and disease free survival (DFS). For OS, death was the clinical endpoint and for DFS occurrence of recurrence, metastasis or death was the clinical endpoint. In statistical analysis, Cox regression method was used to estimate the hazard ratios (HRs) and 95% confidence intervals (CIs) together with p values. After the initial univariate analysis, genetic markers were also investigated in the multivariable analyses adjusting for clinicopathological factors; clinicopathological features with significant p values (<0.05) in the univariate analyses were entered in the multivariable models containing the genetic variables. Since vascular and lymphatic invasion data were highly correlated (~97% of the patients had the same status for both invasions), we entered only the vascular invasion in the multivariable models. Patients who did not experience the outcome of interest were censored at the time of last follow-up. Statistical significance threshold was set at p = 0.05 and all tests were double-sided. All statistical analyses were done using the SPSS statistical software (IBM version 19, USA), unless stated otherwise.

## Results

### Description of the patient cohorts

In this study, two sub-cohorts of the NFCCR patient cohort were investigated: (1) 536 patients investigated for the six mtDNA polymorphisms, and (2) 276 patients investigated for the mtDNA copy number change. The baseline characteristics of these sub-cohorts are shown in Table 1. The median OS and DFS times were 6.3 and 5.98 years in the 536 patient cohort and 6.2 and 5.1 years in the 276 patient cohort (Table 1). There were no significant differences between the NFCCR cohort and the sub-cohort investigated for the relative mtDNA copy number in terms of their baseline characteristics (*data not shown*). However, when compared to the NFCCR cohort, the sub-cohort analyzed for polymorphisms had less stage IV patients (p < 0.001), less patients with vascular invasion (p = 0.014), and, less patients with lymphatic invasion (p = 0.037).

### Survival analysis for the six mtDNA polymorphisms

The genotype frequencies of the six mtDNA polymorphisms are shown in Additional file 1: Table S1.

In the univariate analysis, none of the six mtDNA polymorphisms were associated with OS or DFS (Table 2). As expected, male sex, increasing stage, tumors with vascular and lymphatic invasions and MSI-L/MSS tumor phenotype were significantly associated with increased risk of death in the OS analysis (Additional file 2: Table S2). Similarly, in the univariate analysis for DFS, patients with tumors in rectum, male sex, tumors with advanced stage, vascular and lymphatic invasions and MSI-L/MSS phenotype were associated with increased risk of recurrence, metastasis or death (Additional file 2: Table S2). Multivariable analyses did not identify associations of these polymorphisms with either OS or DFS, either (Additional file 3: Table S3). None of the polymorphism genotypes were associated with the clinicopathological characteristics, though for the 10398 (A/G) polymorphism and the MSI status a borderline *p* value (p = 0.05) was detected (Additional file 4: Table S4).

**Table 2:** Univariate analysis results for the six mtDNA polymorphisms investigated

Variables	n	p value	HR	95% CI		Variables	n	p value	HR	95% CI	
				Lower	Upper					Lower	Upper
Overall survival						Disease free survival					
MitoT479C (C vs *T*)	529	0.451	0.731	0.323	1.652	MitoT479C (C vs *T*)	528	0.917	0.965	0.495	1.884
MitoT491C (C vs *T*)	503	0.631	1.169	0.617	2.215	MitoT491C (C vs *T*)	502	0.182	1.466	0.835	2.574
MitoT10035C (C vs *T*)	534	0.916	0.968	0.526	1.781	MitoT10035C (C vs *T*)	533	0.970	1.011	0.577	1.772
MitoA13781G (G vs *A*)	522	0.734	0.895	0.473	1.695	MitoA13781G (G vs *A*)	521	0.834	0.940	0.524	1.684
10398 (G vs *A*)	528	0.596	0.895	0.594	1.349	10398 (G vs *A*)	527	0.946	1.013	0.698	1.470
16189 (C vs *T*)	528	0.794	0.940	0.590	1.497	16189 (C vs *T*)	527	0.535	0.872	0.565	1.345

Reference categories are italicized.

*CI* confidence interval, *HR* hazard ratio, *n* number of patients, *vs* versus.

### Survival analysis for the mtDNA copy number ratio

**Figure 1:**
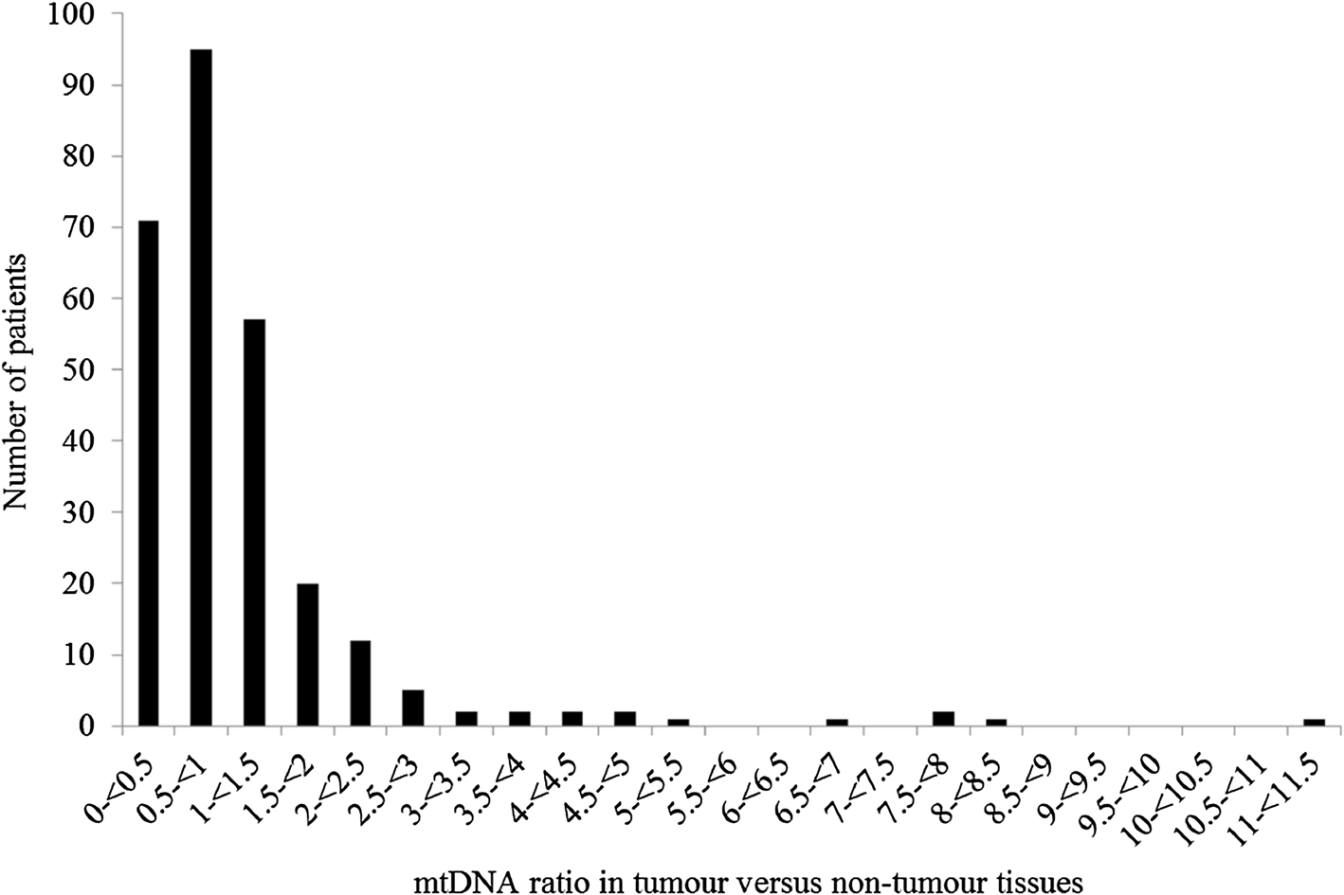
The mtDNA ratio in tumor tissue versus non-tumor tissues in the patients.

Results of the mtDNA copy number analysis obtained in 274 of 276 colorectal cancer patients are summarized in Figure 1. In this patient cohort, the ratio of mtDNA in tumor to non-tumor tissue (mtDNA_T/N_) varied between 0.003 and 11. The majority (94.8%) of the mtDNA_T/N_ were less than 3. In rare cases, a considerable increase in tumor mtDNA was observed (patients with mtDNA_T/N_ ratios ≥3–11, n = 14). An inspection of the clinicopathological features of these 14 patients showed a tendency towards having tumors with stage III and IV (10 out of 14), with MSS/MSI-L (13 out of 14) and with no *BRAF1*-Val600Glu mutation (13 out of 14; the remaining patient’s *BRAF1* mutation status was not determined).

Overall, in 108 patients (39.4%) an increase (mtDNA_T/N_ >1) and in 166 patients (60.6%) a decrease (mtDNA_T/N_ <1) of the mtDNA copy number was detected in tumors with respect to unaffected tissues (Figure 1).

The univariate analysis results for OS and DFS using the mtDNA copy number ratio are summarized in Table 3. As a result, no association of the mtDNA copy number change with patient outcome was detected. Multivariable analysis did not change these results (Additional file 3: Table S3). In addition, we checked the association of the mtDNA copy number change with the baseline characteristics of the patients; although there was a trend for association with stage (p = 0.075) and microsatellite instability status (p = 0.074), no statistically significant association was detected between the mtDNA copy number ratio and the patient baseline characteristics shown in Table 1 (Additional file 4: Table S4). The statistical analysis results for the clinicopathological features with OS and DFS in this sub-cohort are provided in Additional file 5: Table S5.

**Table 3:** Univariate analysis results for the mtDNA copy number change in tumor tissues with respect to non-tumor tissues

Variable	n	p value	HR	95% CI		Variable	n	p value	HR	95% CI	
				Lower	Upper					Lower	Upper
Overall survival						Disease free survival					
mtDNA copy number (increase vs *decrease*)	273	0.389	1.163	0.825	1.640	mtDNA copy number (increase vs *decrease*)	272	0.389	1.154	0.833	1.601

Reference categories are italicized.

*CI* confidence interval, *HR* hazard ratio, *n* number of patients, *vs* versus.

As an exploratory analysis, mtDNA_T/N_ was re-categorized into two groups following a more stringent definition criterion: patients with the mtDNA_T/N_ <0.8 (n = 131) were categorized as the patients with reduced mtDNA copy number and the patients with mtDNA_T/N_ >1.2 (n = 82) were categorized as the patients with increased mtDNA copy number. Similar to our previous result, in this analysis as well there was no evidence of association of the mtDNA copy number change with OS or DFS in colorectal cancer (Additional file 6: Table S6).

## Discussion

Identifying biomarkers that can predict the outcome in cancer patients is a hot topic in cancer research. Mitochondrial dysfunction has been linked to the initiation and progression of cancers [22–24]. Thus, mitochondrial DNA variations are biologically relevant, potential prognostic markers in cancer. In this study we have analyzed the associations of overall and disease-free survival times with the genotypes of six mtDNA polymorphisms [MitoT479C, MitoT491C, MitoT10035C, MitoA13781G, 16189 (T/C) and 10398 (A/G)] in 536 patients and the mtDNA copy number ratio in tumor tissues to non-tumor tissues in 274 patients. Our results suggest that none of these mtDNA variations are linked to prognostic characteristics of colorectal cancer patients.

### MtDNA polymorphisms and their relation to prognosis in colorectal cancer

The mtDNA D-loop region contains the initiation sites for the mtDNA replication and transcription, thus variations in this region may influence these important mitochondrial processes. We studied three polymorphisms located in the D-loop: MitoT491C, MitoT479C and mtDNA 16189 (T/C). There is no report in literature studying the biological effects of the MitoT491C and the MitoT479C polymorphisms, but the 16189 (T/C) polymorphism has been studied by several groups. Briefly, the mtDNA 16189 (T/C) polymorphism is located in a hyper-variable region in the mitochondrial D-loop. This substitution leads to the formation of a homopolymeric C-tract between positions 16189–16194, resulting in a mtDNA length heteroplasmy [7]. This heteroplasmic length variation has been suggested to affect the mtDNA replication [8]. In addition, this polymorphism has been associated with increased oxidative damage [25]. These findings suggest a role for the 16189 T > C polymorphism in increased rate of somatic mutations in mtDNA and altered mitochondrial function, which makes it a good candidate as a prognostic marker in cancer. However, in this study association of this polymorphism with clinical outcome in colorectal cancer was not detected.

Two other polymorphisms investigated in this study are located in the genes coding for the OXPHOS proteins: the 10398 (A/G) is a non-synonymous substitution (Thr114Ala) located in the *ND*-*3* gene and the MitoA13781G is a non-synonymous substitution (Ile482Val) located in the *ND*-*5* gene [26, 27]. Of these two polymorphisms, the biological consequence of 10398 (A/G) has been reported in literature. Kulawiec et al. showed that the cybrid cell lines that had mtDNAs with the A-allele of this polymorphism had elevated levels of reactive oxygen species [6]. Also, these cybrid cell lines showed increased colony forming ability and promoted metastases in mice. Therefore, the 10398 (A/G) polymorphism has several carcinogenesis and progression related effects, yet our results do not support a role of this polymorphism in progression of colorectal cancer.

Similar to other polymorphisms investigated in this study, we did not identify an association of the MitoT10035C polymorphism with overall and disease free survivals in colorectal cancer patients. This polymorphism is located in a mitochondrial tRNA gene and whether or not it causes an alteration in mitochondrial function is currently unknown.

In contrast to polymorphisms from the nuclear DNA genes, mtDNA polymorphisms and their associations with prognosis in colorectal cancer patients have been rarely studied [28, 29]. In one of these studies, the MitoT479C, MitoT491C, MitoT10035C, MitoA13781G and 10398 (A/G) polymorphisms were not found to be associated with outcome in a large Scottish colorectal cancer cohort [29]. Hence, the results that we obtained in our study are concordant with these published results. Yet to our knowledge, this is the first time that the association of the 16189 (T/C) polymorphism with outcome was investigated in colorectal cancer.

### MtDNA copy number change and its relation to prognosis in colorectal cancer

The number of mitochondria may increase or decrease in normal tissues based on the energy requirements of the cells. In cancer, the altered energy needs of the tumor cells and the somatic mutations in mtDNA are likely to contribute to changes in the copy number of mitochondria (thus, the copy number of mtDNA) [9, 13, 23]. In fact, differences in copies of mtDNA in tumor tissues when compared to surrounding unaffected tissues have been shown in various cancers, such as ovarian [30], breast [31], and colorectal cancers [9–13, 32].

In colorectal cancer, associations of the mtDNA copy number change with clinicopathological features, notably stage and outcome, were also reported [10–12, 32]. While the differences in experimental approaches and the cut-off value used to define the relative mtDNA copy number make an objective comparison of these studies difficult, they are nevertheless briefly discussed here. Feng et al. [12] showed that except one patient, the quantity of mtDNA was higher in tumors compared to adjacent non-tumor tissues in 44 patients from China and this increase was associated with the disease stage. In contrast, Cui et al. [32] reported that the 70% of the 60 Chinese colorectal cancer patients had decreased mtDNA copy number in tumor tissues compared to the non-tumor tissue. When they used a cut-off threshold of <0.72 to define a decreased mtDNA copy number, these authors did find an association of mtDNA copy number with the lymph-node metastasis in the univariate analyses, but not with 3-year overall survival and the clinicopathological features including the stage. Lin and co-authors, on the other hand, reported a two-fold increase of mtDNA copy number in tumors of 39% of 153 colorectal cancer patients from Taiwan [10]. These authors also found that the decrease in mtDNA content in tumor tissue when compared to normal tissue was associated with shorter 5-year overall survival (and advanced stage) in the univariate but not in multivariable analysis. In the same study, an association with 5-year disease-free survival was not detected [10]. Presumably the same group in another study reported the mtDNA copy number change in 194 colorectal cancer patients also from Taiwan [11]. In this study, the mtDNA copy numbers were considered to be decreased if the estimated mtDNA quantity in the tumor tissue was less than 2/3 of the mtDNA quantity in the unaffected tissue. This study showed a decreased tumor mtDNA copy number in 28% of the patients and associations of the decreased mtDNA copy number with stage and reduced 5-year disease-free survival in univariate analysis, but not in multivariable analysis [11].

In our analysis, we observed an increase or decrease of the mtDNA copy number in tumor tissues of ~39 and ~61% of the patients, respectively (Figure 1), however we did not find any association between the mtDNA copy number change status and overall and disease-free survivals in our cohort (Table 3; Additional file 3: Table S3). Using a stringent threshold to define the increase and decrease in relative mtDNA copy number did not change these results (Additional file 6: Table S6). We also did not identify statistically significant associations between the baseline characteristics, including stage, and the mtDNA copy number change status in our patient cohort (Additional file 4: Table S4). Our results therefore contradict the previously published results showing the association of the mtDNA copy number ratio with clinicopathological or outcome characteristics in colorectal cancer patients [10–12, 32]. This discrepancy in results can be attributed to the differences between these studies and ours (such as analyzing cohorts with different follow-up periods, ethnicities, baseline clinicopathological or tumor molecular characteristics) in addition to the differences in study designs (such as the application of different cut-off values to define the increase or decrease of the relative mtDNA copy number). Clearly, based on ours and previously published results, a consistent conclusion on the degree of the change of the mtDNA copy number in colorectal tumors and its association with patient clinicopathological features and clinical outcome cannot be made. Further studies are needed to elucidate whether or not the tumor mtDNA copy number change is a prognostic marker in colorectal cancer.

A limitation of our study was that the NFCCR patient sub-cohort genotyped for the polymorphisms was biased towards earlier stages when compared to the entire NFCCR patient cohort. This bias should be kept in mind while interpreting our results. On the other hand, it is also one of the few fairly large colorectal cancer cohorts investigated for prognostic associations of mtDNA polymorphisms. In addition, to our knowledge this is the first study that investigated the mtDNA 16189 (T/C) polymorphism and survival characteristics of the colorectal cancer patients. This is also the first time the mtDNA copy number change was investigated in relation to clinical outcome in Caucasian colorectal cancer patients. While this NFCCR patient sub-cohort is characterized by a relatively long-follow up period and is also unique in being the largest cohort studied till date (n = 274), we cannot rule out the possibility that our sample size may not be large enough to detect a possible association of the mtDNA copy number change with outcome.

## Conclusions

Overall, comprehensive studies on large patient cohorts are required to identify the potential prognostic roles of the mtDNA variations investigated in this study and the survival outcomes of colorectal cancer patients.

## Additional files

Additional file 1:
**Table S1**. Details of the TaqMan^®^ SNP genotyping and qPCR reactions and the genotype frequencies of the mtDNA polymorphisms investigated. Additional file 2:
**Table S2.** Results of the univariate analyses for the clinicopathological features (SNP genotyped cohort). Additional file 3:
**Table S3.** Multivariable analysis results for the six mtDNA SNPs and the mtDNA copy number change. Additional file 4:
**Table S4.** Chi square or Fisher exact test results for the mtDNA polymorphisms and copy number ratio and the clinicopathological features. Additional file 5:
**Table S5.** Results of the univariate analyses for the clinicopathological features (qPCR cohort). Additional file 6:
**Table S6.** Univariate analysis for the secondary mtDNA copy number ratio classification.

## Abbreviations

CI: confidence interval
C_T_: the cycle number that bisects the threshold line
DFS: disease-free survival
HR: hazards ratio
HREB: human research ethics board
MAF: minor allele frequency
MSI: microsatellite instability
MSI-H: microsatellite instability-high
MSI-L: microsatellite instability-low
MSS: microsatellite stable
mtDNA: mitochondrial DNA
mtDNA_T/N_: the ratio of mtDNA copy number in the tumor tissue to non-tumor tissue
ND3: NADH dehydrogenase 3
ND5: NADH dehydrogenase 5
NFCCR: Newfoundland colorectal cancer registry
OS: overall survival
OXPHOS: oxidative phosphorylation pathway
SDS: sequence detection system
vs: versus
qPCR: quantitative polymerase chain reaction
