# Supplementary material for: Mitochondrial DNA polymorphisms, its copy number change and outcome in colorectal cancer
Source: BMC Res Notes. 2015 Jun 27;8:272. doi: 10.1186/s13104-015-1250-5 (PMC4482280; doi:10.1186/s13104-015-1250-5)
Supplement: Additional file 1: — Table S1. Details of the TaqMan® SNP genotyping and qPCR reactions and the genotype frequencies of the mtDNA polymorphisms investigated. [file 13104_2015_1250_MOESM1_ESM.pdf]

Additional File 1: Details of the TaqMan® SNP genotyping and qPCR reactions and the genotype frequencies of the mtDNA polymorphisms investigated

a) Primer and probe information for the mtDNA 16189 (T/C) polymorphism

**Primers**	***TaqMan® probes**
**Forward primer**<br>CACCTGTAGTACATAAAAACCCAATCCA	**VIC probe (T allele)**<br>CCCCCTCCCCATGCT
**Reverse primer**<br>GGGTTGATTGCTGTACTTGCTTGTA	**FAM probe (C allele)**<br>CCCCCCCCCATGCT

*Polymorphic alleles are underlined. Applied Biosystem assay ID: AHKA OSD.

b) Genotype frequencies for the six mtDNA polymorphisms investigated in this study

Polymorphism	rs number	Major allele, homoplasmic genotype n (%)	Minor allele, homoplasmic genotype n (%)	*Heteroplasmic genotype, n (%)	Number of patients with missing genotypes data (%)
10398 (A/G)	rs2853826	A, 443 (82.6)	G, 86 (16.0)	AG, 1 (0.19)	6 (1.1)
16189 (T/C)	rs55749223	T, 463 (86.4)	C, 66 (12.3)	TC, 1 (0.19)	6 (1.1)
MitoT479C	rs41442247	T, 505 (94.2)	C, 25 (4.7)	0	6 (1.1)
MitoT491C	rs28625645	T, 477 (89)	C, 27 (5.0)	0	32 (6)
MitoT10035C	rs41347846	T, 500 (93.3)	C, 35 (6.53)	0	1 (0.19)
MitoA13781G	rs41358152	A, 489 (91.2)	G, 34 (6.3)	0	13 (2.4)

*heteroplasmy happens when a proportion of mtDNA copies carry one allele and the others carry the variant allele.

c) The contents of the qPCR 20X TaqMan® assay mix (for 50 µl).

**Reagent**	**Volume (µl)**	
	**mtDNA (ND-2)**	**nDNA (FASLG)**
Forward primer (100 µM)	2.25	18
Reverse primer (100 µM)	2.25	18
Probe (100 µM)	0.5	4
Sub-total	5	40
1X TE buffer	5	
**Total**	**50**	
