# Supplementary material for: Mitochondrial DNA polymorphisms, its copy number change and outcome in colorectal cancer
Source: BMC Res Notes. 2015 Jun 27;8:272. doi: 10.1186/s13104-015-1250-5 (PMC4482280; doi:10.1186/s13104-015-1250-5)
Supplement: Additional file 2: — Table S2. Results of the univariate analyses for the clinicopathological features (SNP genotyped cohort). [file 13104_2015_1250_MOESM2_ESM.pdf]

Additional File 2: Results of the univariate analyses for the clinicopathological features (SNP genotyped cohort)

a) overall survival

Variable	n	p-value	HR	95% CI for HR Lower	95% CI for HR Upper
Sex (male vs female)	535	**0.011**	1.499	1.096	2.050
Histology (mucinous vs non-mucinous)	535	0.889	0.968	0.608	1.540
Location (rectum vs colon)	535	0.201	1.216	0.901	1.639
Stage	535	**<0.001**			
Stage (II vs I)		0.176	1.456	0.845	2.507
Stage (III vs I)		**0.004**	2.215	1.299	3.779
Stage (IV vs I)		**<0.001**	10.142	5.770	17.825
Grade (poorly differentiated/undifferentiated vs well/moderately differentiated	531	0.667	0.875	0.475	1.610
Vascular invasion (+ vs -)	496	**<0.001**	1.717	1.269	2.324
Lymphatic invasion (+ vs -)	493	**0.003**	1.575	1.166	2.127
Familial risk (high/moderate vs low)	535	0.627	1.075	0.803	1.440
MSI status (MSI-H vs MSS/MSI-L)	513	**<0.001**	0.233	0.103	0.526
BRAF Val600Glu mutation status (+ vs -)	484	0.398	0.795	0.467	1.353
Age	535	0.414	1.007	0.991	1.023

(+): presence, (-): absence, CI: Confidence Interval, HR: Hazard Ratio, MSI-H: Microsatellite Instability-High, MSI-L: Microsatellite instability-Low, MSS: Microsatellite Stable, n: number of patients. P-values <0.05 are shown in bold.

b) disease free survival

Variable	n	p-value	HR	95% CI for HR Lower	Upper
Sex (male vs female)	534	**0.008**	1.479	1.107	1.975
Histology (mucinous vs non-mucinous)	534	0.775	0.939	0.609	1.448
Location (rectum vs colon)	534	**0.031**	1.354	1.028	1.783
Stage	534	**<0.001**			
Stage (II vs I)		0.244	1.327	0.825	2.133
Stage (III vs I)		**0.001**	2.138	1.343	3.405
Stage (IV vs I)		**<0.001**	5.739	3.459	9.520
Grade (poorly differentiated/undifferentiated vs well/moderately differentiated	530	0.481	0.811	0.453	1.452
Vascular invasion (+ vs -)	495	**0.001**	1.637	1.236	2.169
Lymphatic invasion (+ vs -)	492	**0.003**	1.528	1.156	2.020
Familial risk (high/moderate vs low)	534	0.275	1.163	0.887	1.524
MSI status (MSI-H vs MSS/MSI-L)	512	**0.001**	0.346	0.183	0.653
BRAF Val600Glu mutation status (+ vs -)	484	0.651	0.896	0.558	1.440
Age	534	0.866	1.001	0.987	1.016

(+): presence, (-): absence, CI: Confidence Interval, HR: Hazard Ratio, MSI-H: Microsatellite Instability-High, MSI-L: Microsatellite instability-Low, MSS: Microsatellite Stable, n: number of patients. P-values <0.05 are shown in bold.
