# Supplementary material for: Mitochondrial DNA polymorphisms, its copy number change and outcome in colorectal cancer
Source: BMC Res Notes. 2015 Jun 27;8:272. doi: 10.1186/s13104-015-1250-5 (PMC4482280; doi:10.1186/s13104-015-1250-5)
Supplement: Additional file 3: — Table S3. Multivariable analysis results for the six mtDNA SNPs and the mtDNA copy number change. [file 13104_2015_1250_MOESM3_ESM.pdf]

Additional File 3: Multivariable analysis results for the six mtDNA SNPs and the mtDNA copy number change

a) Overall survival and mtDNA polymorphisms

10398

			95% CI for HR		
**Variables**	**p-value**	**HR**	**Lower**	**Upper**	**n**
10398 (G vs A)	0.921	0.978	0.626	1.528	472
Sex (male vs female)	0.101	1.333	0.945	1.879	
Stage	**<0.001**				
Stage (II vs I)	0.140	1.531	.869	2.696	
Stage (III vs I)	**0.034**	1.878	1.050	3.361	
Stage (IV vs I)	**<0.001**	8.896	4.829	16.388	
Vascular invasion (+ vs -)	0.346	1.175	0.840	1.645	
MSI status (MSI-H vs MSS/MSI-L)	**0.001**	0.197	0.072	0.536	

16189

			95% CI for HR		
**Variables**	**p-value**	**HR**	**Lower**	**Upper**	**n**
16189 (C vs T)	0.832	0.948	0.578	1.555	472
Sex (male vs female)	0.096	1.339	0.950	1.887	
Stage	**<0.001**				
Stage (II vs I)	0.144	1.524	0.866	2.684	
Stage (III vs I)	**0.035**	1.870	1.046	3.344	
Stage (IV vs I)	**<0.001**	8.852	4.807	16.302	
Vascular invasion (+ vs -)	0.319	1.187	0.848	1.660	
MSI status (MSI-H vs MSS/MSI-L)	**0.001**	0.197	0.072	0.534	

MitoA13781G

Variables	p-value	HR	95% CI for HR Lower	Upper	n
MitoA13781G (G vs A)	0.986	0.994	0.485	2.034	467
Sex (male vs female)	0.113	1.319	0.936	1.858	
Stage	**<0.001**				
Stage (II vs I)	0.187	1.466	0.831	2.588	
Stage (III vs I)	**0.048**	1.800	1.005	3.224	
Stage (IV vs I)	**<0.001**	8.491	4.602	15.668	
Vascular invasion (+ vs -)	0.170	1.265	0.904	1.770	
MSI status (MSI-H vs MSS/MSI-L)	**0.004**	0.298	0.130	0.683	

MitoT479C

Variables	p-value	HR	95% CI for HR Lower	Upper	n
MitoT479C (C vs T)	0.662	0.818	0.333	2.010	472
Sex (male vs female)	0.106	1.321	0.943	1.849	
Stage	**<0.001**				
Stage (II vs I)	0.097	1.632	0.915	2.911	
Stage (III vs I)	**0.021**	2.016	1.114	3.647	
Stage (IV vs I)	**<0.001**	9.077	4.866	16.933	
Vascular invasion (+ vs -)	0.286	1.198	0.859	1.670	
MSI status (MSI-H vs MSS/MSI-L)	**0.005**	0.309	0.135	0.703	

MitoT491C

Variables	p-value	HR	95% CI for HR Lower	Upper	n
MitoT491C (C vs T)	0.356	1.380	0.697	2.734	449
Sex (male vs female)	**0.044**	1.428	1.010	2.019	
Stage	**<0.001**				
Stage (II vs I)	0.158	1.522	0.849	2.727	

Stage (III vs I)	**0.028**	1.948	1.074	3.534	
Stage (IV vs I)	**<0.001**	8.644	4.630	16.138	
Vascular invasion (+ vs -)	0.234	1.227	0.876	1.719	
MSI status (MSI-H vs MSS/MSI-L)	**0.006**	0.315	0.137	0.722	

MitoT10035C

			95% CI for HR		
**Variables**	**p-value**	**HR**	**Lower**	**Upper**	**n**
MitoT10035C (C vs T)	0.959	0.981	0.480	2.008	477
Sex (male vs female)	0.093	1.334	0.953	1.867	
Stage	**<0.001**				
Stage (II vs I)	0.130	1.548	0.880	2.723	
Stage (III vs I)	**0.031**	1.891	1.059	3.375	
Stage (IV vs I)	**<0.001**	8.891	4.842	16.326	
Vascular invasion (+ vs -)	0.273	1.203	0.864	1.676	
MSI status (MSI-H vs MSS/MSI-L)	**0.004**	0.294	0.129	0.673	

b) Disease free survival and polymorphisms

10398

			95% CI for HR		
**Variables**	**p-value**	**HR**	**Lower**	**Upper**	**n**
10398 (G vs A)	0.844	1.041	0.698	1.553	471
Sex (male vs female)	0.145	1.265	0.922	1.736	
Location (rectum vs colon)	0.138	1.263	0.928	1.719	
Stage	**<0.001**				
Stage (II vs I)	0.180	1.402	0.855	2.300	
Stage (III vs I)	**0.021**	1.817	1.094	3.019	

Stage (IV vs I)	**<0.001**	5.340	3.044	9.370	
Vascular invasion (+ vs -)	0.446	1.130	0.825	1.547	
MSI status (MSI-H vs MSS/MSI-L)	**0.007**	0.369	0.179	0.762	

16189

			95% CI for HR		
**Variables**	**p-value**	**HR**	**Lower**	**Upper**	**n**
16189 (C vs T)	0.495	0.850	0.532	1.357	471
Sex (male vs female)	0.134	1.274	0.928	1.748	
Location (rectum vs colon)	0.129	1.270	0.933	1.728	
Stage	**<0.001**				
Stage (II vs I)	0.180	1.402	0.856	2.297	
Stage (III vs I)	**0.021**	1.818	1.096	3.015	
Stage (IV vs I)	**<0.001**	5.345	3.051	9.361	
Vascular invasion (+ vs -)	0.421	1.137	0.831	1.557	
MSI status (MSI-H vs MSS/MSI-L)	**0.008**	0.373	0.181	0.769	

MitoA13781G

			95% CI for HR		
**Variables**	**p-value**	**HR**	**Lower**	**Upper**	**n**
MitoA13781G (G vs A)	0.962	0.985	0.518	1.871	466
Sex (male vs female)	0.149	1.262	0.920	1.730	
Location (rectum vs colon)	0.219	1.215	0.891	1.656	
Stage	**<0.001**				
Stage (II vs I)	0.228	1.356	0.826	2.225	
Stage (III vs I)	**0.033**	1.736	1.045	2.884	
Stage (IV vs I)	**<0.001**	5.134	2.925	9.012	
Vascular invasion (+ vs -)	0.234	1.210	0.884	1.655	
MSI status (MSI-H vs MSS/MSI-L)	0.021	0.462	0.239	0.892	

MitoT479C

			95% CI for HR		
**Variables**	**p-value**	**HR**	**Lower**	**Upper**	**n**
MitoT479C (C vs T)	0.767	1.114	0.545	2.279	471
Sex (male vs female)	0.171	1.243	0.910	1.697	
Location (rectum vs colon)	0.136	1.263	0.929	1.718	
Stage	**<0.001**				
Stage (II vs I)	0.119	1.489	0.903	2.458	
Stage (III vs I)	**0.017**	1.874	1.120	3.135	
Stage (IV vs I)	**<0.001**	5.562	3.152	9.814	
Vascular invasion (+ vs -)	0.318	1.172	0.858	1.600	
MSI status (MSI-H vs MSS/MSI-L)	**0.014**	0.424	0.214	0.842	

MitoT491C

			95% CI for HR		
**Variables**	**p-value**	**HR**	**Lower**	**Upper**	**n**
MitoT491C (C vs T)	0.158	1.532	0.847	2.772	448
Sex (male vs female)	0.101	1.307	0.949	1.800	
Location (rectum vs colon)	0.135	1.273	0.928	1.746	
Stage	**<0.001**				
Stage (II vs I)	0.264	1.336	0.804	2.221	
Stage (III vs I)	**0.026**	1.804	1.075	3.029	
Stage (IV vs I)	**<0.001**	5.254	2.976	9.277	
Vascular invasion (+ vs -)	0.311	1.179	0.858	1.620	
MSI status (MSI-H vs MSS/MSI-L)	**0.024**	0.453	0.228	0.902	

MitoT10035C

			95% CI for HR		
**Variables**	**p-value**	**HR**	**Lower**	**Upper**	**n**
MitoT10035C (C vs T)	0.941	0.976	0.514	1.853	476

Sex (male vs female)	0.127	1.274	0.933	1.738
Location (rectum vs colon)	0.143	1.257	0.926	1.706
Stage	**<0.001**			
Stage (II vs I)	0.163	1.419	0.867	2.323
Stage (III vs I)	**0.020**	1.818	1.097	3.013
Stage (IV vs I)	**<0.001**	5.384	3.082	9.404
Vascular invasion (+ vs -)	0.369	1.153	0.846	1.571
MSI status (MSI-H vs MSS/MSI-L)	**0.022**	0.463	0.240	0.893

c) Overall and disease free survivals for mtDNA copy number change

mtDNA_copy_number-overall survival

			95% CI		
	**p-value**	**HR**	**Lower**	**Upper**	**n**
Stage	**<0.001**				245
Stage II versus I	0.560	1.339	0.501	3.579	
Stage III versus I	0.088	2.319	0.883	6.095	
Stage IV versus I	**<0.001**	14.606	5.420	39.357	
Grade (poorly differentiated/undifferentiated vs well/moderately differentiated)	**0.048**	1.727	1.005	2.969	
Vascular invasion (+ versus -)	**0.004**	1.781	1.209	2.625	
MSI status (MSI-H versus MSS/MSI-L)	**0.003**	0.164	0.050	0.538	
Age	**0.001**	1.038	1.016	1.060	
mtDNA_copy_number (increase versus decrease)	0.551	0.890	0.606	1.306	

mtDNA_copy_number-disease free survival

			95% CI		
	**p-value**	**HR**	**Lower**	**Upper**	**n**

Stage	**<0.001**				225
Stage II versus I	0.813	1.109	0.472	2.602	
Stage III versus I	0.268	1.614	0.692	3.763	
Stage IV versus I	**<0.001**	6.576	2.711	15.953	
Grade (poorly differentiated/undifferentiated vs well/moderately differentiated)	0.551	1.201	0.659	2.188	
Vascular invasion (+ versus -)	**0.005**	1.753	1.184	2.596	
MSI status (MSI-H versus MSS/MSI-L)	**0.025**	0.359	0.146	0.881	
mtDNA_copy_number (increase versus decrease)	0.957	1.010	0.690	1.480	
Sex (female versus male)	0.268	1.236	0.849	1.799	
BRAF Val600Glu mutation (+ versus -)	**0.014**	1.990	1.150	3.442	

P-values <0.05 are shown in bold.
