# Supplementary material for: Mitochondrial DNA polymorphisms, its copy number change and outcome in colorectal cancer
Source: BMC Res Notes. 2015 Jun 27;8:272. doi: 10.1186/s13104-015-1250-5 (PMC4482280; doi:10.1186/s13104-015-1250-5)
Supplement: Additional file 4: — Table S4. Chi square or Fisher exact test results for the mtDNA polymorphisms and copy number ratio and the clinicopathological features. [file 13104_2015_1250_MOESM4_ESM.pdf]

Additional File 4: Chi-square or Fisher exact test results for the mtDNA polymorphisms and copy number ratio and the clinicopathological features.

10398

**Variables compared**	**p-value**
10398 G/A * Sex	.518
10398 G/A * Histology	.442
10398 G/A * Location	.440
10398 G/A * Stage	.914
10398 G/A * Grade	.573
10398 G/A * Vascular_invasion	.951
10398 G/A * Lymphatic_invasion	.973
10398 G/A * Familial_risk	.198
10398 G/A * MSI_status	**.050**
10398 G/A * BRAF_status	.355

16189

**Variables compared**	**p-value**
16189 T/C * Sex	.487
16189 T/C * Histology	.538
16189 T/C * Location	.458
16189 T/C * Stage	.994
16189 T/C * Grade	.126
16189 T/C * Vascular_invasion	.462
16189 T/C * Lymphatic_invasion	.294
16189 T/C * Familial_risk	.730
16189 T/C * MSI_status	.543
16189 T/C * BRAF_status	.955

MitoA13781G

**Variables compared**	**p-value**
MitoA13781G * Sex	.962
MitoA13781G * Histology	.251
MitoA13781G * Location	.556
MitoA13781G * Stage	.859
MitoA13781G * Grade	1.000
MitoA13781G * Vascular_invasion	.961
MitoA13781G * Lymphatic_invasion	.897
MitoA13781G * Familial_risk	.791
MitoA13781G * MSI_status	.068
MitoA13781G * BRAF_status	.540

MitoT479C

**Variables compared**	**p-value**

MitoT479C * Sex	.921
MitoT479C * Histology	.755
MitoT479C * Location	.532
MitoT479C * Stage	.361
MitoT479C * Grade	.700
MitoT479C * Vascular_invasion	.793
MitoT479C * Lymphatic_invasion	.565
MitoT479C * Familial_risk	.662
MitoT479C * MSI_status	.739
MitoT479C * BRAF_status	1.000

MitoT491C

**Variables compared**	**p-value**
MitoT491C * Sex	.310
MitoT491C * Histology	.756
MitoT491C * Location	.079
MitoT491C * Stage	.782
MitoT491C * Grade	.711
MitoT491C * Vascular_invasion	.396
MitoT491C * Lymphatic_invasion	.325
MitoT491C * Familial_risk	.462
MitoT491C * MSI_status	.058
MitoT491C * BRAF_status	1.000

MitoT10035C

**Variables compared**	**p-value**
MitoT10035C * Sex	.888
MitoT10035C * Histology	.270
MitoT10035C * Location	.756
MitoT10035C * Stage	.808
MitoT10035C * Grade	1.000
MitoT10035C * Vascular_invasion	.813
MitoT10035C * Lymphatic_invasion	.684
MitoT10035C * Familial_risk	.661
MitoT10035C * MSI_status	.066
MitoT10035C * BRAF_status	.541

mtDNA copy number change

**Variables compared**	**p-value**
mtDNA copy number * Sex	0.352
mtDNA copy number * Histology	0.998
mtDNA copy number * Location	0.34
mtDNA copy number * Stage	0.075
mtDNA copy number * Grade	0.306

mtDNA copy number * Vascular invasion	0.935
mtDNA copy number * Lymphatic invasion	0.977
mtDNA copy number * Familial risk	0.754
mtDNA copy number * MSI status	0.074
mtDNA copy number * BRAF status	0.148
