# Supplementary material for: Mitochondrial DNA polymorphisms, its copy number change and outcome in colorectal cancer
Source: BMC Res Notes. 2015 Jun 27;8:272. doi: 10.1186/s13104-015-1250-5 (PMC4482280; doi:10.1186/s13104-015-1250-5)
Supplement: Additional file 5: — Table S5. Results of the univariate analyses for the clinicopathological features (qPCR cohort). [file 13104_2015_1250_MOESM5_ESM.pdf]

Additional File 5: Results of the univariate analyses for the clinicopathological features (qPCR cohort)

a) overall survival

**Variables compared**	**n**	**p-value**	**HR**	**95 % CI**	
				**Lower**	**Upper**
Sex (male vs female)	273	0.055	1.412	0.993	2.009
Histology (mucinous vs non-mucinous)	273	0.730	0.910	0.531	1.557
Location (rectum vs colon)	273	0.376	0.850	0.594	1.217
Stage	273	**<0.001**			
Stage (II vs I)		0.297	1.597	0.663	3.847
Stage (III vs I)		**<0.001**	3.065	1.314	7.150
Stage (IV vs 1)		**<0.001**	17.45	7.385	41.25
Grade (poorly differentiated/undifferentiated vs well/moderately differentiated)	272	**0.019**	1.796	1.103	2.926
Vascular invasion (+ vs -)	247	**<0.001**	2.187	1.527	3.131
Lymphatic invasion (+ vs -)	247	**<0.001**	1.937	1.355	2.770
Familial risk (high/moderate vs low)	272	0.973	1.006	0.715	1.415
MSI status (MSI-H vs MSS/MSI-L)	271	**0.004**	0.230	0.085	0.623
BRAF1 Val600Glu mutation status (+ vs -)	251	0.195	1.374	0.850	2.220
Age	273	**0.025**	1.023	1.003	1.043

(+): presence, (-): absence, CI: Confidence Interval, HR: Hazard Ratio, MSI-H: Microsatellite Instability-High, MSI-L: Microsatellite Instability-Low, MSS: Microsatellite Stable, n: number of patients. P-values <0.05 are shown in bold.

b) disease free survival

**Variables compared**	**n**	**p-value**	**HR**	**95% CI**	
				**Lower**	**Upper**
Sex (male vs female)	272	**0.046**	1.41	1.007	1.964
Histology (mucinous vs non-mucinous)	272	0.757	0.923	0.557	1.531
Location (rectum vs colon)	272	0.969	0.993	0.709	1.393
Stage	272	**<0.001**			
Stage (II vs I)		0.324	1.474	0.682	3.185
Stage (III vs I)		**0.006**	2.850	1.360	5.985
Stage (IV vs 1)		**<0.001**	11	5.155	23.558
Grade (poorly differentiated/undifferentiated vs well/moderately differentiated)	271	**0.029**	1.69	1.055	2.716
Vascular invasion (+ vs -)	246	**<0.001**	2.05	1.457	2.877
Lymphatic invasion (+ vs -)	246	**<0.001**	1.88	1.34	2.644
Familial risk (high/moderate vs low)	271	0.567	1.099	0.795	1.520
MSI status (MSI-H vs MSS/MSI-L)	270	**0.014**	0.390	0.181	0.826
BRAF1 Val600Glu mutation status (+ vs -)	251	**0.049**	1.560	1.003	2.421
Age	272	0.103	1.015	0.997	1.034

(+): presence, (-): absence, CI: Confidence Interval, HR: Hazard Ratio, MSI-H: Microsatellite Instability-High, MSI-L: Microsatellite Instability-Low, MSS: Microsatellite Stable, n: number of patients. P-values <0.05 are shown in bold.
