# Supplementary material for: Mitochondrial DNA polymorphisms, its copy number change and outcome in colorectal cancer
Source: BMC Res Notes. 2015 Jun 27;8:272. doi: 10.1186/s13104-015-1250-5 (PMC4482280; doi:10.1186/s13104-015-1250-5)
Supplement: Additional file 6: — Table S6. Univariate analysis for the secondary mtDNA copy number ratio classification. [file 13104_2015_1250_MOESM6_ESM.pdf]

Additional File 6: Univariate analysis for the secondary mtDNA copy number ratio classification

Variable	n	p-value	HR	95 % CI Lower	Upper	Variable	n	p-value	HR	95 % CI Lower	Upper
**Overall survival**						**Disease free survival**					
mtDNA copy number (>1.2 vs <0.8)	213	0.414	1.181	0.793	1.759	mtDNA copy number (>1.2 vs <0.8)	212	0.264	1.241	0.850	1.813

CI: Confidence Interval, HR: Hazard Ratio, n: number of patients in the statistical analysis. In this analysis, the mtDNA ratios >1.2 are assumed to represent the increased and <0.8 are assumed to represent the decreased mtDNA quantity in the tumor tissues compared to non-tumor tissues.
